# Simultaneous tuning of the magnetic anisotropy and thermal stability of $\alpha ^{''}$-phase Fe$_{16}$N$_{2}$

**DOI:** 10.1038/s41598-021-87077-2

**Published:** 2021-04-09

**Authors:** D. Odkhuu, S. C. Hong

**Affiliations:** 1 Department of Physics, Incheon National University, Incheon, 22012 South Korea; 2 Department of Physics, University of Ulsan, Ulsan, 44610 South Korea

**Keywords:** Magnetic properties and materials, Electronic structure

## Abstract

Simultaneously enhancing the uniaxial magnetic anisotropy ($K_u$) and thermal stability of $\alpha ^{''}$-phase Fe$_{16}$N$_{2}$ without inclusion of heavy-metal or rare-earth (RE) elements has been a challenge over the years. Herein, through first-principles calculations and rigid-band analysis, significant enhancement of $K_u$ is proposed to be achievable through excess valence electrons in the Fe$_{16}$N$_{2}$ unit cell. We demonstrate a persistent increase in $K_u$ up to 1.8 MJ m$^{\text {-}3}$, a value three times that of 0.6 MJ m$^{\text {-}3}$ in $\alpha ^{''}$-Fe$_{16}$N$_{2}$, by simply replacing Fe with metal elements with more valence electrons (Co to Ga in the periodic table). A similar rigid-band argument is further adopted to reveal an extremely large $K_u$ up to 2.4 MJ m$^{\text {-}3}$ in (Fe$_{0.5}$Co$_{0.5}$)$_{16}$N$_{2}$ obtained by replacing Co with Ni to Ga. Such a strong $K_u$ can also be achieved with the replacement by Al, which is isoelectronic to Ga, with simultaneous improvement of the phase stability. These results provide an instructive guideline for simultaneous manipulation of $K_u$ and the thermal stability in 3*d*-only metals for RE-free permanent magnet applications.

## Introduction

Alpha-phase iron has been known for its extraordinary magnetic properties, including high saturation magnetization ($\mu _0 M_s$) and Curie temperature ($T_c$), in addition to its relatively simple fabrication and low price. These intriguing features make it a potential champion ever for high-performance permanent magnet applications^1–4^. However, the main drawback of $\alpha$-Fe is its negligible uniaxial magnetic anisotropy ($K_u$) on the order of $\mu$eV per atom^1–4^. Two practical approaches to enhance $K_u$ in $\alpha$-Fe are (1) alloying with heavy-metal (HM) or rare-earth (RE) elements, with the most prominent examples being FePt^5^ and Nd$_2$Fe$_{14}$B^6, 7^, and (2) reducing the crystal symmetry from the cubic ($c/a=1$) to tetragonal phase ($c/a\ne 1$)^8–10^. In (1), 5*d* or 4*f* electrons possess inherently large spin-orbit coupling (SOC) and orbital angular momentum (*L*), but the inclusion of these HM and RE elements is not desirable in terms of price and is detrimental to $\mu _0 M_s$ and $T_c$. In (2), the energy levels of the 3*d* orbitals evolve in tetragonal symmetry, particularly around the Fermi level ($E_F$), which in turn enhances $K_u$^11^.

The tetragonal phase of $c/a\ne 1$ is now accessible in epitaxial Fe films with a diverse choice of lattice-mismatched substrates. Nevertheless, such tetragonal distortion is feasible only for limited film thicknesses of a few nanometers^12, 13^. In contrast, a bulk-scale tetragonal structure of the $\alpha ^{''}$-phase (*c*/*a* $=$ 1.1) is favored when 12.5 at.% N is embedded into the $\alpha$-Fe structure with octahedral interstitial sites, forming a 16:2 (Fe$_{16}$N$_2$) stoichiometry^14^. Since a surprisingly large magnetic moment of 2.6−3 $\mu _B$ per Fe atom was reported^15–17^, $\alpha ^{''}$-phase Fe$_{16}$N$_2$ has received enormous attention as a possible 3*d*-only permanent magnet. However, the practical implementation of $\alpha ^{''}$-Fe$_{16}$N$_2$ in obtaining monophasic samples is quite difficult as $\alpha ^{''}$-Fe$_{16}$N$_2$ decomposes into $\alpha$-Fe and $\gamma ^\prime$-Fe$_4$N at a low temperature of approximately 500 K^14^. Numerous efforts have been made to improve the thermal stability of $\alpha ^{''}$-Fe$_{16}$N$_2$; the most successful approach is Ti addition but the magnetic properties are greatly suppressed^18, 19^. In addition to the weak thermal stability, another major obstacle that hampers practical applications is the still insufficient $K_u$, which ranges from 0.4 to 1 MJ m$^{\text {-}3}$, depending on the sample preparation and film thickness^20–22^. In the research community, search for enhancing $K_u$ while improving the thermal stability of $\alpha ^{''}$-Fe$_{16}$N$_{2}$ in the bulk has been thus very intensive and remains unresolved.

In this article, we propose a possible mechanism of tuning the number of valence electrons to simultaneously enhance the thermal stability and $K_u$ by a few times in Fe$_{16}$N$_{2}$ and (Fe$_{0.5}$Co$_{0.5}$)$_{16}$N$_{2}$ apart from the aforementioned approaches (1) and (2), using first-principles calculations and rigid-band model analysis. We predict a persistent increase in $K_u$ up to 2.4 MJ m$^{\text {-}3}$, which is four times that (0.6 MJ m$^{\text {-}3}$) of Fe$_{16}$N$_{2}$, by replacing Fe with metal elements with more valence electrons (Co to Ga and Al in the periodic table). Such a supreme $K_u$ is discussed in connection with the mutual mechanisms of the Jahn−Teller orbital splitting and excess electron-induced energy level changes in the electronic structure.

## Results and discussion

**Figure 1:**
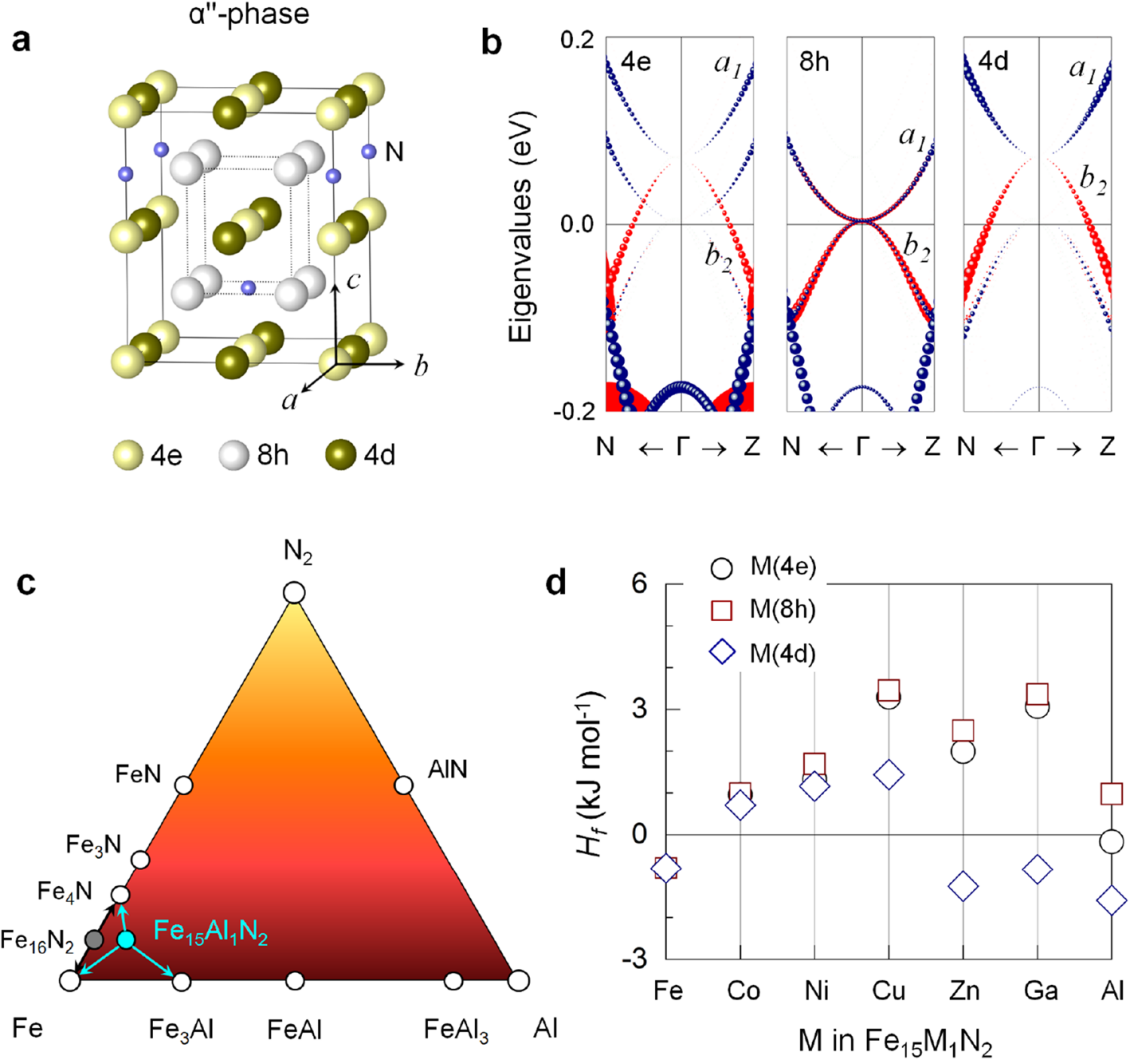
(**a**) Crystal structure of $\alpha ^{''}$-phase Fe$_{16}$N$_{2}$. (**b**) Spin-down channel eigenvalues of the $a_1$ and $b_2$ orbitals of Fe(4e), Fe(8h), and Fe(4d) along the high-symmetry N$\Gamma$Z line of the Brillouin zone. The size of the symbols is proportional to their weights. The Fermi level is set to zero. (**c**) Schematic phase diagram of the ternary Fe−Al−N system. (**d**) Enthalpy of formation $H_f$ of Fe$_{15}$M$_1$N$_{2}$ (M $=$ Co−Ga and Al) for M(4e), M(8h), and M(4d).

Figure 1a displays the $\alpha ^{''}$-phase structure of Fe$_{16}$N$_{2}$. The optimized *a* and *c*/*a* are 5.69 Å and 1.1, respectively. The corresponding values in an experiment are 5.72 Å and 1.1^14^. In the unit cell, 16 Fe atoms occupy 3 inequivalent sites at the Wyckoff positions of 4e, 8h, and 4d, while 2 N are at the octahedral interstices with the 4 Fe(8h) coordination^14^. These 4e, 8h, and 4d sites differ in magnetic moment (Table 1): 2.14 (2.33), 2.36 (2.45), and 2.82 (3.05) $\mu _B$ in the present theory (experiment^17^), respectively. The mechanism is associated with the nonidentical Fe–N bond lengths: Fe(4e)–N: 1.83, Fe(8h)–N: 1.95, and Fe(4d)–N: 3.24 Å. Furthermore, the energy levels of their 3*d* orbitals evolve near $E_F$, particularly in the spin-down channel (Fig. 1b).

**Table 1:** Optimized tetragonal distortion *c*/*a*, magnetic moments ($\mu _B$) of Fe(4e), Fe(8h), Fe(4d), and M-replacement elements, saturation magnetization $\mu _0 M_s$ (T), anisotropic field $\mu _0 H_a$ (T), hardness parameter $\kappa$, and Curie temperature $T_c$ (K) of Fe$_{15}$M$_1$N$_{2}$ for M $=$ Fe−Ga and Al.

	*c*/*a*	Magnetic Moment ($\mu _B$)				$\mu _0 M_s$ (T)	$\mu _0 H_a$ (T)	$\kappa$	$T_c$ (K)
		Fe(4e)	Fe(8h)	Fe(4d)	M(4d)				
Fe	1.10	2.14	2.36	2.82	2.82	2.24	0.65	0.38	925
Co	1.10	2.15	2.43	2.82	1.93	2.22	0.94	0.45	.
Ni	1.11	2.10	2.38	2.77	0.82	2.12	0.71	0.41	.
Cu	1.11	2.08	2.34	2.72	0.10	2.04	0.82	0.44	.
Zn	1.11	2.10	2.29	2.74	-0.08	2.01	1.58	0.62	760
Ga	1.11	2.11	2.22	2.76	-0.14	1.98	2.35	0.77	804
Al	1.11	2.11	2.21	2.76	-0.07	1.98	2.24	0.75	855

Only one Fe atom in the 16 Fe unit cell is replaced by a 3*d*-metal atom (M $=$ Co−Ga and Al), which corresponds to approximately 5.5 at.% doping. For each M, we have considered three different substitution sites, i.e., M(4e), M(8h), and M(4d). The $\alpha ^{''}$-phase stability upon M replacement can be inspected by the enthalpy of formation: $H_f = (H - \sum _i \mu _i N_i)N_A/N$, where $\mu _i$ and $N_i$ are the chemical potential and number of decomposable components *i*, respectively. $N_A$ and *N* are the Avogadro constant and the number of atoms in the unit cell. The obtained $H_f$ values of Fe$_{16}$N$_{2}$ are  -3.14 kJmol$^{\text {-}1}$ against ($\alpha$-Fe)+N$_2$ and  -0.80 kJ mol$^{\text {-}1}$ against ($\alpha$-Fe)+($\gamma ^\prime$-Fe$_4$N) decomposition. The small negative value of the latter implies that the $\alpha ^{''}$-Fe$_{16}$N$_{2}$ phase is stable at a low temperature but most likely decomposes into the $\alpha$-Fe and $\gamma ^\prime$-Fe$_4$N phases at an elevated temperature, as observed experimentally^14^. From the ternary Fe−M−N phase diagram, as an example for M $=$ Al in Fig. 1c, Fe$_4$N and Fe$_3$M phases are identified as the most competitive binary decomposable phases to Fe$_{15}$M$_1$N$_2$. For M $=$ Ni (Cu and Zn), Fe$_4$N+FeNi(Cu/Zn)+Fe decomposition has been considered since Fe$_3$Ni (Fe$_3$Cu/Zn and FeCu/Zn) is unstable.

**Figure 2:**
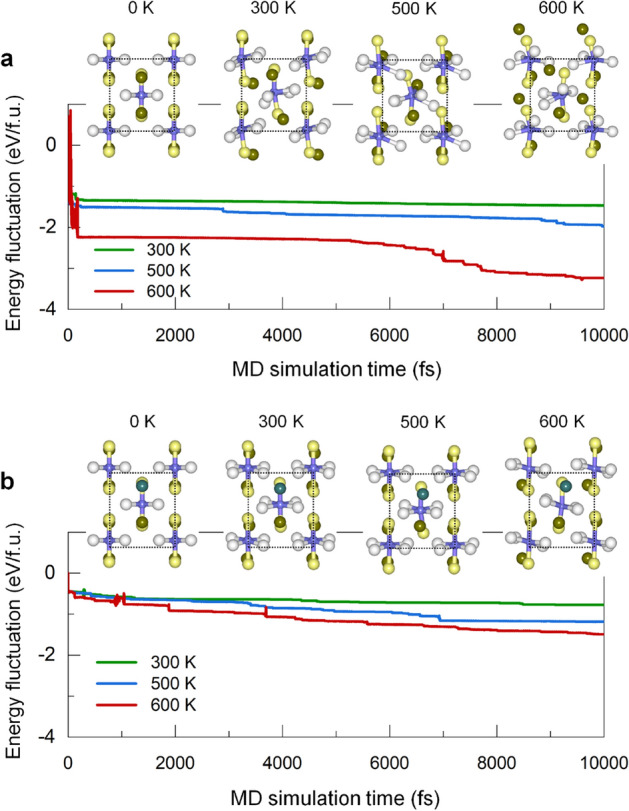
AIMD simulation of the free energy fluctuation of (**a**) Fe$_{16}$N$_2$ and (**b**) Fe$_{15}$Al$_1$N$_2$ for given temperatures 300, 500, and 600 K. The insets show the side views of the corresponding atomic structures before (0 K) and after the AIMD simulation period of 10 ps. The atomic symbols are the same as used in Fig. 1a. Green sphere in **b** is the Al atom.

Figure 1d presents the calculated $H_f$ of Fe$_{15}$M$_1$N$_2$ (M $=$ Co−Ga and Al) for M(4e), M(8h), and M(4d). All the M elements prefer 4d-site replacement, which in turn splits the neighboring Fe(4d) sites into Fe(4d)$_c$ along the *c* axis and Fe(4d)$_{ab}$ on the *ab* plane with dissimilar magnetic properties, as addressed in the following paragraphs. The $\alpha ^{''}$-phase becomes unstable upon Co to Cu replacements. In contrast, the replacement of Zn, Ga, and Al improves the $\alpha ^{''}$-phase stability with $H_f$ enhanced by 0.04−0.8 kJ mol$^{\text {-}1}$ in magnitude, as their nitrides (ZnN, GaN, and AlN) have higher standard enthalpies of formation, in the range of  -100 to  -320 kJ mol$^{\text {-}1}$^23–25^, than FeN (-47 kJ mol$^{\text {-}1}$)^25^. The completely filled *d*-orbitals of the Zn and Ga elements provide extra stability to the system, as these elements have a symmetrical distribution of electrons and larger exchange energies than Fe^26^.

We further investigate the structural stability at an elevated temperature using *ab initio* molecular dynamic (AIMD) simulation. Figure 2a,b present the fluctuations of the total free energy of the selected Fe$_{16}$N$_{2}$ and Fe$_{15}$Al$_1$N$_2$ phases for given temperatures 300, 500, and 600 K, respectively. The total energy of Fe$_{16}$N$_{2}$ decreases immediately within a few fs by 1.3 (at 300 K)−2.2 eV/f.u. (at 600 K), which is associated with the thermal motion and relocation of atomic coordinates. Furthermore, the energy variation during the AIMD simulation increases with temperature and reaches 3.2 eV/f.u. at 600 K, where the $\alpha ^{''}$-phase structure is largely distorted, as indicated in the insets in Fig. 2a. On the other hand, the energy fluctuation of Fe$_{15}$Al$_1$N$_2$ phase is rather small within 1.5 eV/f.u. even at 600 K (Fig. 2b). In particular, the $\alpha ^{''}$ phase tends to maintain up to 600 K (insets in Fig. 2b), although marginal phonon vibrations and atomic coordinate distortions occur during the AIMD simulation.

**Figure 3:**
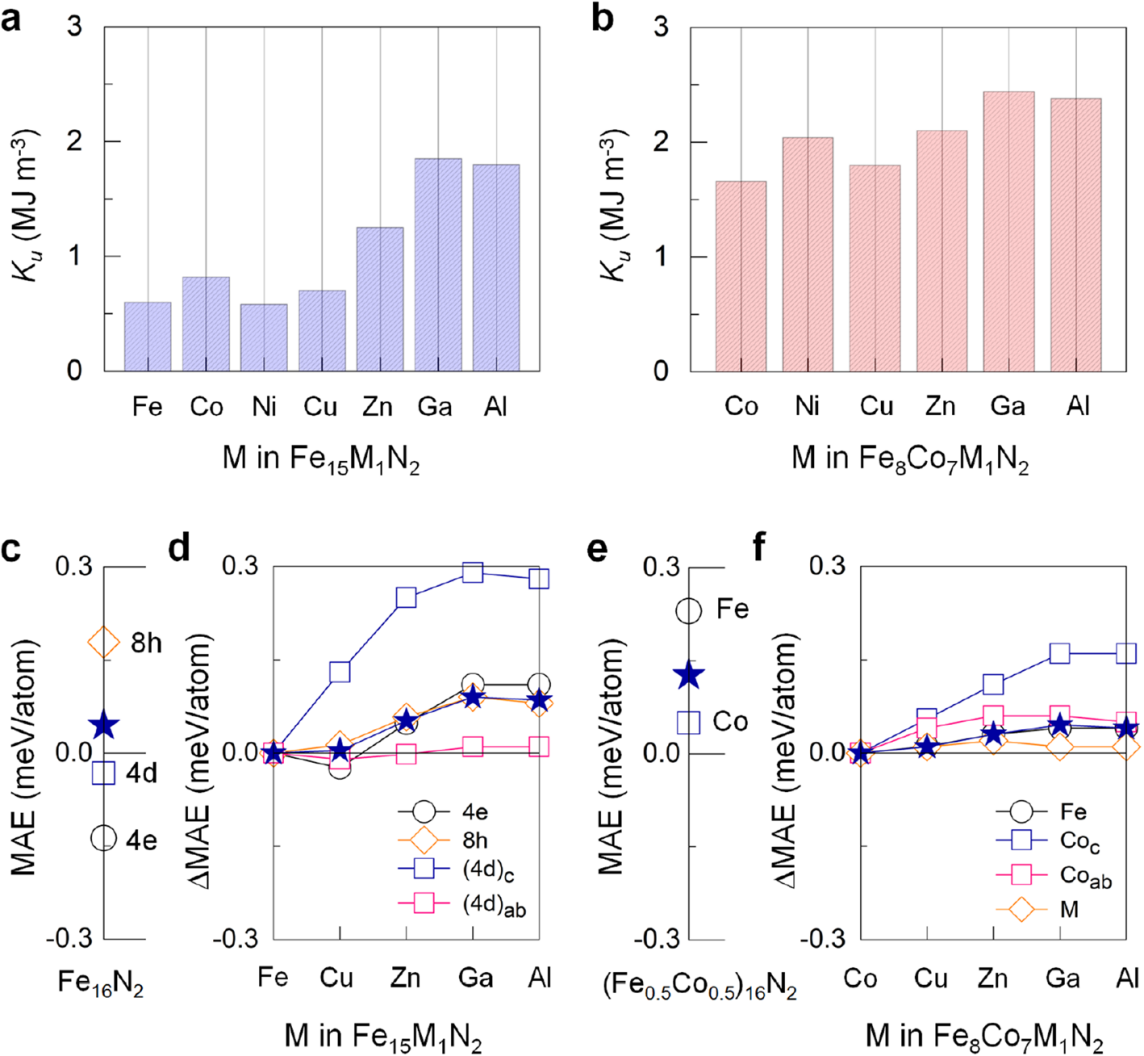
Predicted uniaxial magnetic anisotropy $K_u$ of (**a**) Fe$_{16}$N$_{2}$ and (**b**) (Fe$_{0.5}$Co$_{0.5}$)$_{16}$N$_{2}$ with M replacement (M $=$ Co−Ga and Al). (**c**,**e**) Atom-decomposed (open symbols) and total (filled star) magnetocrystalline anisotropy energy MAE, (**d**,**f**) M (Cu−Ga and Al)-induced enhancement in MAE ($\Delta \text {MAE}$) of Fe$_{16}$N$_2$ and (Fe$_{0.5}$Co$_{0.5}$)$_{16}$N$_2$.

In Fig. 3a, an even more notable finding is the persistent increase in $K_u$ as M changes from Ni to Ga, reaching the largest value of 1.85 MJ m$^{\text {-}3}$ for Ga replacement. This value is more than 3 times the enhancement attained for $\alpha ^{''}$-Fe$_{16}$N$_{2}$. The present $K_u$ of Fe$_{16}$N$_{2}$ is 0.6 MJ m$^{\text {-}3}$, which is within the range of experimental values of 0.4−1 MJ m$^{\text {-}3}$^20–22^. The enhanced $K_u$ of the $\alpha ^{''}$-phase is associated with tetragonal distortion. The Jahn−Teller-like *d*-orbital level splitting when $\alpha \rightarrow \alpha ^{''}$ offers more electronic energy level degrees of freedom^10^. More specifically, the tetragonal distortion splits the cubic $e_g$ and $t_{2g}$ levels around $E_F$: singlets $a_1$ ($d_{x^2\text {-}y^2}$) and $b_1$ ($d_{3r^2\text {-}z^2}$), and singlet $b_2$ ($d_{xy}$) and doublet *e* ($d_{yz,xz}$), respectively. Evidently, the energy levels near $E_F$ of the spin-down electrons, especially the $a_1$ and $b_2$ states, differ at the 4e, 8h, and 4d sites (Fig. 1b). Further analyses indicate that the difference comes from their dissimilar hybridization with N-2*p* orbitals.

**Figure 4:**
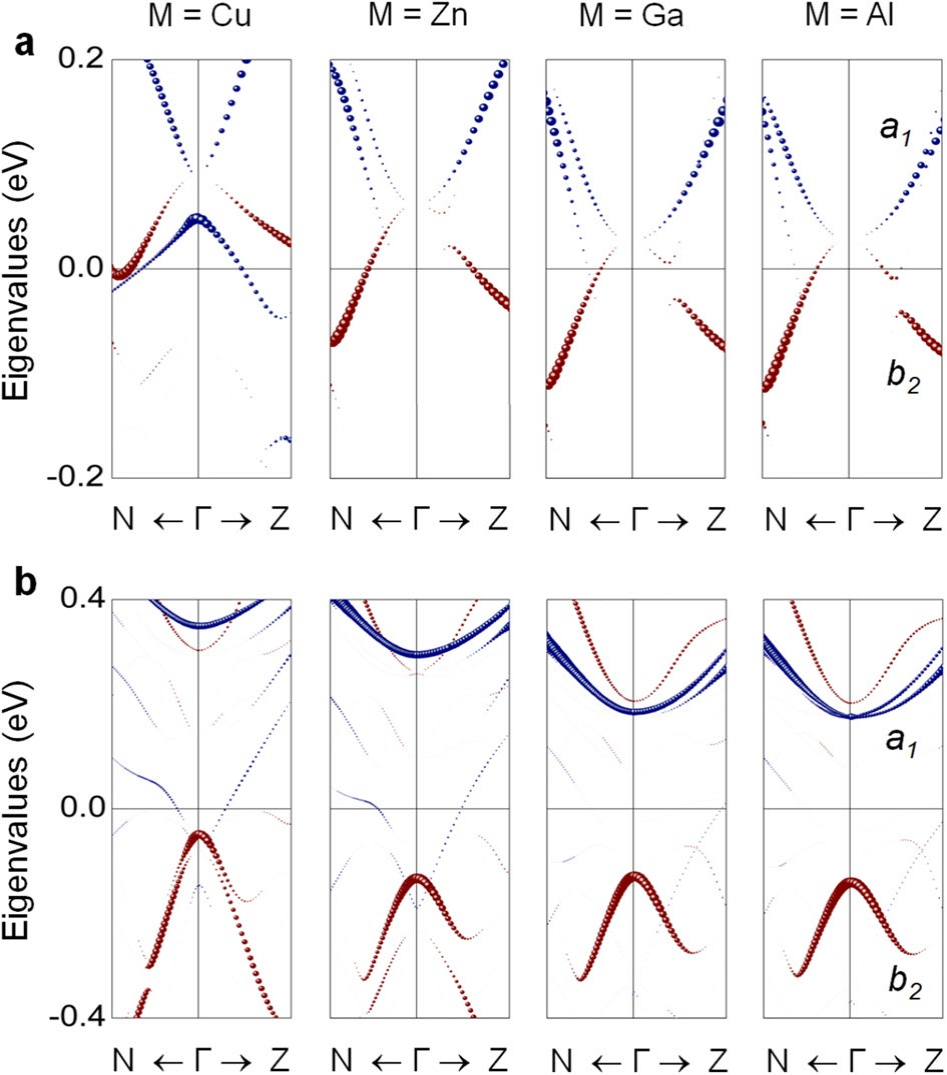
Spin-down channel eigenvalues of $a_1$ (blue) and $b_2$ (red) orbitals of the (**a**) Fe(4d)$_c$ atom in M-replaced Fe$_{16}$N$_{2}$ and (**b**) Co$_c$ atom in M-replaced (Fe$_{0.5}$Co$_{0.5}$)$_{16}$N$_{2}$ along the high-symmetry N$\Gamma$Z line of the Brillouin zone for M $=$ Cu−Ga and Al. The size of the symbols is proportional to their weights. The Fermi level is set to zero.

Our analysis of atom resolved magnetocrystalline anisotropy energy (MAE) in Fig. 3c indicates that MAE in this $\alpha ^{''}$-phase distributes unequally over the unit cell: -0.14, 0.18, and -0.03 meV at the 4e, 8h, and 4d sites, respectively. Here, MAE is scaled down to the microscopic atomic level (meV/atom), rather than the macroscopic energy density (MJ m$^{\text {-}3}$). From the *k*-resolved (minority-spin) eigenvalue analysis in Fig. 1b, featured bands with cone-like shapes occur: the minimum of $a_1$ (parabolic) and maximum of $b_2$ (reverse parabolic) dispersions touch at the $\Gamma$ point. In particular, for Fe(8h), such a conical $a_1-b_2$ pair appears right at $E_F$. In association with their reduced eigenvalue difference across $E_F$, the SOC matrix term in the Hamiltonian can thus increase the positive contribution to MAE, according to the perturbation theory^11^.

According to Fig. 3d, the Fe(4d)$_c$ site plays a major role in the M(Cu to Ga)-induced enhancement in MAE ($\Delta \text {MAE}$) rather than the Fe(4d)$_{ab}$ site. The contributions to MAE from the other 4e and 8h sites cannot be ignored although minor. Meanwhile, the conical $a_1-b_2$ pair of the Fe(4d)$_c$ site moves gradually toward $E_F$ with the Cu to Ga replacement (Fig. 4a), which reflects the rigid-band model. A similar phenomenon is not present for the Fe(4d)$_{ab}$ site because of its longer separation (4 Å) from M than Fe(4d)$_c$ (3.1 Å). We therefore attribute MAE in Fe$_{15}$M$_1$N$_2$ to the joint effects of the Jahn−Teller level splitting and the supplied-electron-induced level changes of the *d*-orbitals.

In the rigid-band picture, the shift of the electronic states is related to the change in the energy of the Bloch state with M ($\Delta \varepsilon _k$), as $\rho (\varepsilon ) = \rho _0(\varepsilon ) - [ \partial \rho _0(\varepsilon _k) /\partial \varepsilon _k ] \Delta \varepsilon _k$^27^, where $\rho (\varepsilon )$ and $\rho _0(\varepsilon )$ are the density of states (DOS) of Fe(4d)$_c$ in Fe$_{15}$M$_1$N$_{2}$ and Fe$_{16}$N$_{2}$, respectively. For a small amount of M, $\Delta \varepsilon _k$ is also small and thus independent of *k*, where the shape of the band structure remains the same but displaced by $\Delta \varepsilon _k$. Eventually, for the Ga replacement, the conical $a_1-b_2$ pair of the Fe(4d)$_c$ site shifts down and appears near $E_F$, which in turn enhances MAE. Here, the Jahn−Teller argument is not applicable, as the *c*/*a* (1.1) of $\alpha ^{''}$-Fe$_{16}$N$_{2}$ remains almost the same upon M replacement (Table 1).

**Figure 5:**
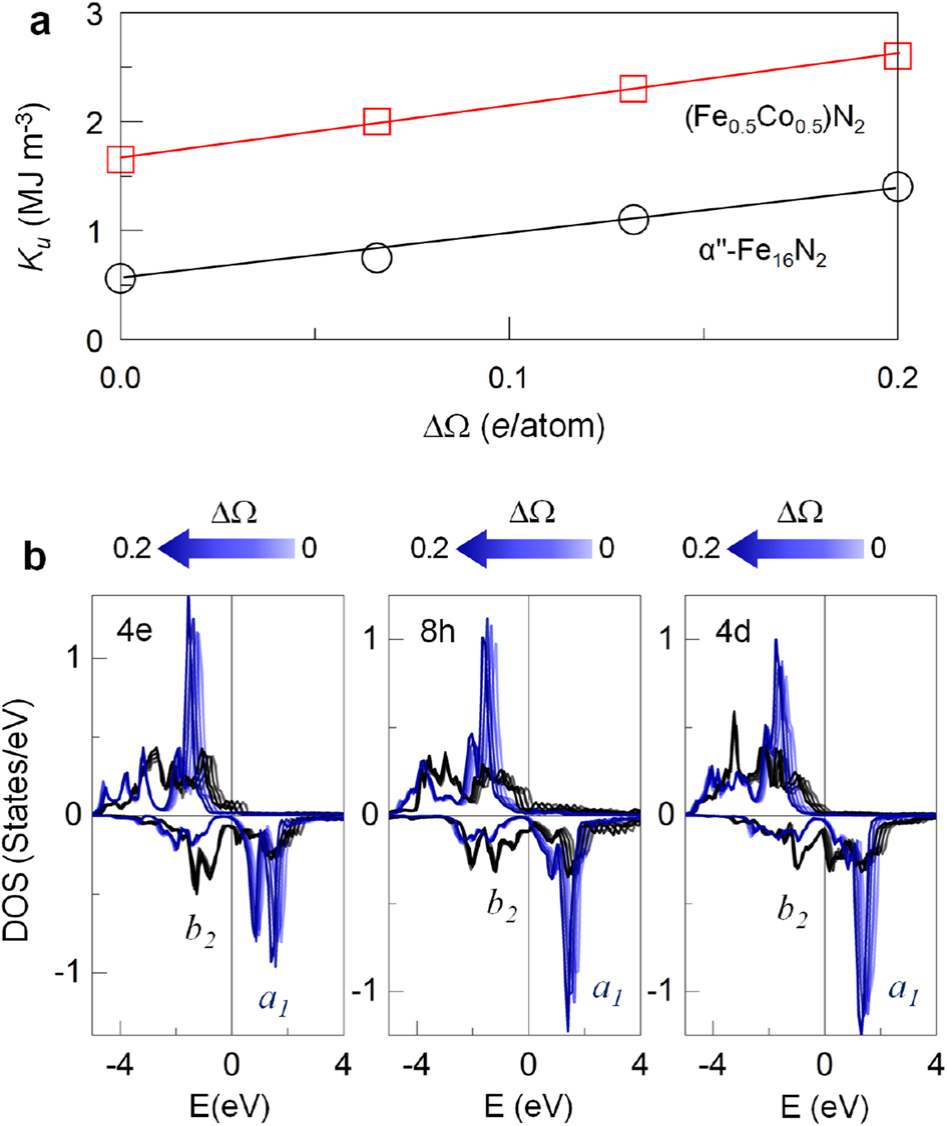
(**a**) Predicted $K_u$ of Fe$_{16}$N$_{2}$ (circle) and (Fe$_{0.5}$Co$_{0.5}$)$_{16}$N$_{2}$ (square) as a function of the number of excess valence electrons, $\Delta \Omega$, in a unit cell. (**b**) $\Delta \Omega$-dependent DOS of the $a_1$ (blue) and $b_2$ (black) orbitals of Fe(4e), Fe(8h), and Fe(4d) of Fe$_{16}$N$_{2}$. The color-scale from light to dark in the DOS corresponds to the enhancement of $\Delta \Omega$ from 0 to 0.2 *e*/atom. The Fermi level is set to zero.

In accordance with the $a_1-b_2$ shift (Fig. 4a), the replacement element that can maximize MAE is Ga, in line with the obtained $K_u$ in Fig. 3a. To support this scenario, we explore the replacement element Al, which is isoelectronic to Ga. Remarkably, we find $K_u$ value of 1.80 MJ m$^{\text {-}3}$ for the Al replacement (Fig. 3a), similar to that (1.85 MJ m$^{\text {-}3}$) for the Ga replacement. Accordingly, similar electronic features of the $a_1-b_2$ bands and $\Delta \varepsilon _k$ at the Fe(4d)$_c$ site are identified in Fig. 4a for the same group elements, Al and Ga. Furthermore, as mentioned early in Fig. 1d, the inclusion of Al in Fe$_{16}$N$_2$ greatly improves the $\alpha ^{''}$-phase stability beyond the other M-replacements. From a practical viewpoint, the Ga and Al replacements (particularly, Al) for Fe are desirable for RE-free permanent magnets because of their abundances on earth.

In line with the argument we outlined thus far, an even larger $K_u$ may be achieved if more Fe in Fe$_{16}$N$_{2}$ are replaced with metal elements with more valence electrons. To test this scenario, we replace half of the Fe in Fe$_{16}$N$_{2}$ with Co. From the total energy minimization, all the 4d and 4e sites are occupied by Co, forming the B2-phase, while 2 N prefer the 4 Fe(8h) coordinated octahedral interstices on the same *ab* plane. As Co has 1 more electron and stronger SOC than Fe, we find that the $K_u$ in (Fe$_{0.5}$Co$_{0.5}$)$_{16}$N$_{2}$ is 1.65 MJ m$^{\text {-}3}$. This value is more than double that (0.6 MJ m$^{\text {-}3}$) of $\alpha ^{''}$-Fe$_{16}$N$_{2}$. A similar argument can be applied for other replacements such as Ni and Zn (not shown here). Furthermore, the enhanced *c*/*a* (1.17) of (Fe$_{0.5}$Co$_{0.5}$)$_{16}$N$_{2}$, compared with 1.1 in Fe$_{16}$N$_{2}$, is clearly an additional cause of the large $K_u$^8^.

Remarkably, the M-replaced (Fe$_{0.5}$Co$_{0.5}$)$_{16}$N$_{2}$ compounds exhibit a trend similar to, but with notably enhanced numerical values, that in Fe$_{16}$N$_{2}$: a nearly linear increase in $K_u$ from Cu to Ga (Fig. 3b). Eventually, Ga replacement leads to a $K_u$ as high as 2.44 MJ m$^{\text {-}3}$. Such supreme value of $K_u$ can also be achieved for Al (2.41 MJ m$^{\text {-}3}$). These values are more than 4 times that of Fe$_{16}$N$_{2}$ and more than half the value of 4.5 MJ m$^{\text {-}3}$ of the typical RE-magnet Nd$_2$Fe$_{14}$B^28^. Similar to in Fe$_{16}$N$_{2}$, Co next to M on the *c* axis (denoted Co$_c$) produces the largest $\Delta \text {MAE}$ compared with other sites (Fig. 3f), although MAE is larger for Fe than for Co (Fig. 3e). At this Co$_c$ site, it is manifested that the main mechanism of enhancing $K_u$ is the displacement of the (unoccupied) $a_1$ band toward $E_F$ as M changes from Cu to Ga and Al in Fig. 4b.

**Figure 6:**
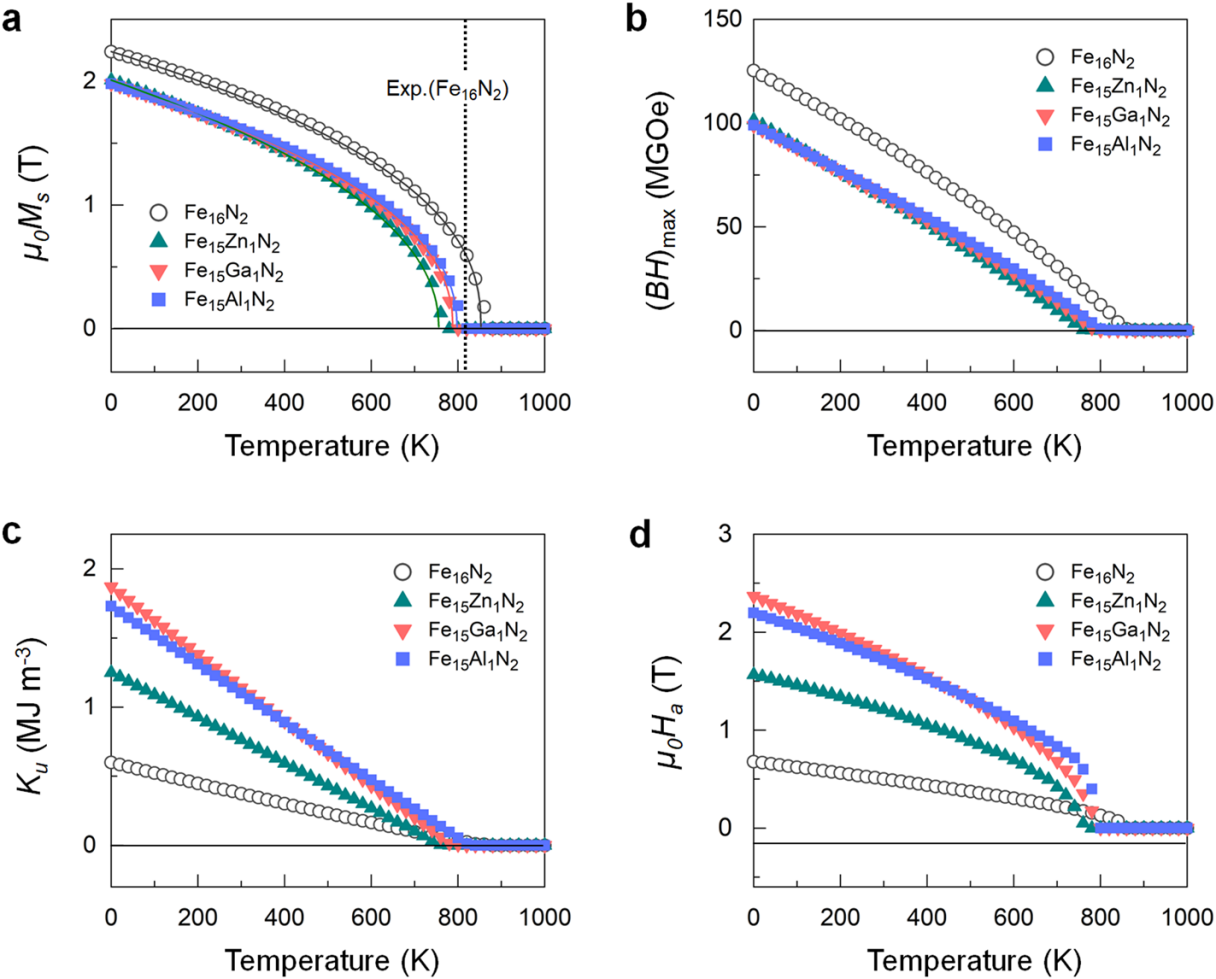
Calculated temperature dependent magnetization of Fe$_{15}$M$_1$N$_{2}$ for M $=$ Fe, Zn, Ga, and Al. The same for $\alpha$-Fe is shown in open circles for reference. The lines are the fitted curves for the magnetization data points. The vertical dotted lines indicate the experimental $T_c$ values of $\alpha$-Fe (1044 K)^31^ and $\alpha ^{''}$-Fe$_{16}$N$_{2}$ (813 K)^32^.

We believe that the present argument is rather general and can be applied to other magnetic materials. To better justify the excess-electron-induced enhancement in $K_u$, we forcibly increase the number of valence electrons in Fe$_{16}$N$_{2}$ and (Fe$_{0.5}$Co$_{0.5}$)$_{16}$N$_{2}$. This approach reflects an excess electron that is uniformly accumulated over all Fe rather than at a specific site neighboring the M replacement. For both Fe$_{16}$N$_{2}$ and (Fe$_{0.5}$Co$_{0.5}$)$_{16}$N$_{2}$, $K_u$ increases linearly as the number of excess electrons ($\Delta \Omega$) increases (Fig. 5a). Nearly the same values of $K_u$ of 1.3 MJ m$^{\text {-}3}$ in Zn-replaced Fe$_{16}$N$_{2}$ and 2.4 MJ m$^{\text {-}3}$ in Ga-replaced (Fe$_{0.5}$Co$_{0.5}$)$_{16}$N$_{2}$ are reproduced at $\Delta \Omega$ $=$ 0.2 *e*/atom. From the simplified DOS analyses in Fig. 5b for Fe$_{16}$N$_{2}$, the unoccupied $a_1$ bands of all the Fe sites displace toward $E_F$ upon an increase in $\Delta \Omega$, while the occupied $b_2$ state is rather insensitive. This result again reveals that the *d*-orbital level change induced by supplied electrons is the main mechanism of the $K_u$ enhancement.

We now would like to highlight the intrinsic hard magnetic properties, including maximum theoretical energy product (*BH*)$_{\text {max}}$, anisotropic field $\mu _0 H_a$, and hardness parameter $\kappa$, of the present compounds. The sufficiently large $\mu _0 M_s$ and thus (*BH*)$_{\text {max}}$, defined as (*BH*)$_{\text {max}}$ $=$ (1/4)$\mu _0 M_s^2$^29^, are worth noting. In Table 1, Fe$_{15}$M$_1$N$_{2}$ exhibits $\mu _0 M_s$ of 2.24−1.98 T, while the (Fe$_{0.5}$Co$_{0.5}$)$_{16}$N$_{2}$-based compounds have a slightly lower magnetization of 1.96−1.80 T. These values are far beyond those of the best high-performance permanent magnets, for example, 1.61 T for Nd$_2$Fe$_{14}$B^28^. The predicted $\mu _0 H_a$ ($=2K_u/M_s$)^29^ increases from 0.65 T for Fe$_{16}$N$_{2}$ to more than 2 (3) T for the Ga/Al-replaced Fe$_{16}$N$_{2}$ ((Fe$_{0.5}$Co$_{0.5}$)$_{16}$N$_{2}$), as shown in Table 1. Additionally, typical permanent magnets possess a hardness parameter $\kappa$ ($=$ ($K_u/\mu _0 M_s^2$)$^{1/2}$) close to or greater than 1^29^. The calculated $\kappa$ of the M-replaced (Fe$_{0.5}$Co$_{0.5}$)$_{16}$N$_{2}$ (M-replaced Fe$_{16}$N$_{2}$) ranges within 0.75−1.10 (0.41−0.78), values which are 2−3 times that (0.38) of Fe$_{16}$N$_{2}$. We finally explore the magnetization dynamics of $\alpha$-Fe and Fe$_{15}$M$_1$N$_{2}$ for the selected M $=$ Fe, Zn, Ga, and Al. From the temperature dependent magnetization in Fig. 6, the absolute value of $T_c$ can be estimated by fitting the magnetization data to the function *M*(*T*) $=$ (1$-T$/$T_c$)$^{\beta }$^30^. We find that the calculated $T_c$ values of $\alpha$-Fe and $\alpha ^{''}$-Fe$_{16}$N$_{2}$ are 1026 and 925 K, respectively, which are in reasonable agreement with the experimental ones (1044 and 813 K)^31, 32^. Furthermore, as listed in Table 1, the obtained $T_c$ values (804−855 K) of the stable compounds (M $=$ Zn, Ga, and Al) are sufficient to fulfill the basic requirement (no less than 550 K) of permanent magnets^33^.

## Conclusion

In summary, we show, using first-principles calculations and rigid-band model analysis of $\alpha ^{''}$-phase Fe$_{16}$N$_{2}$, that $K_u$ can be scaled up by a few times upon the substitution of metal elements with more valence electrons than Fe, from Co to Ga, without the inclusion of RE and HM elements. More remarkably, the replacement by simple metals (Al and Ga) has potential for simultaneous enhancements of $K_u$ and the thermal stability, which would make $\alpha ^{''}$-Fe$_{16}$N$_{2}$ a possibly RE-free permanent magnet, along with its high Curie temperature and low materials price. Furthermore, we demonstrate that a similar argument, as a general rule, is applicable to suitable systems to achieve enhanced intrinsic hard magnetic properties and improved thermal stability. We hope that our results can be used as a guideline for subsequent experimental investigations of RE-free high-performance permanent magnetic materials.

## Methods

The density-functional theory (DFT) calculations were performed using the projector augmented wave (PAW) method^34^, as implemented in the Vienna *ab initio* simulation package (VASP)^35^. The exchange-correlation interactions are treated with the generalized gradient approximation of Perdew, Burke, and Ernzerhof (PBE)^36^. We used an energy cutoff of 500 eV and a $11 \times 11\times$11 Brillouin zone (BZ) *k*-point mesh to relax the lattice parameters and atomic coordinates until the largest force decreased to below 10$^{\text {-}2}$ eV/Å. The total energy method is applied to obtain $K_u$, which is expressed as $K_u = (E_a - E_c)/volume$, where $E_a$ and $E_c$ are the total energies with magnetization along the *a* and *c* axes, respectively. To obtain well-converged $K_u$, we impose a denser *k*-point mesh of $15 \times 15\times$15 with a smaller smearing of 0.05 in the Gaussian method, where the convergence of $K_u$ with respect to the number of *k* points and smearing parameter is ensured. In tetragonal symmetry, $K_u$ is expressed as $K_u \approx K_1 \text {sin}^2 \theta + K_2 \text {sin}^4 \theta$, where $K_1$ and $K_2$ are the magnetic anisotropy constants and $\theta$ is the polar angle between the magnetization vector and the easy axis (*c* axis in the present system). For $\theta = \pi /2$, $K_u = K_1 + K_2$. It is a formidable task to ensure numerical results of $K_u$ with all electron methods, if we start from scratch. To this end, we have also performed full-potential calculations using the WIEN2K package^37^, adopting the optimized lattice constants and ionic positions obtained from the VASP calculations. The two methods produce consistent results. In the AIMD simulation, we adopted the Nosé-thermostat algorithm to model a canonical ensemble^38^. A time step of 1 fs and 10000 ionic steps were used for the total simulation time of 10 ps with the $\Gamma$-point BZ integration, where the lattice parameters and atomic coordinates are allowed to relax at constant volume. The numerical calculations for magnetization dynamics and $T_c$ were carried out using Monte Carlo simulation based on the Heisenberg model in the VAMPIRE package^30^. Here, the Heisenberg spin Hamiltonian is defined by

1 $$\begin{aligned} H = -\frac{1}{2} \sum _{i\ne j}J_{ij}{} \mathbf{S} _i\cdot \mathbf{S} _j-K_u\sum _{i}(\mathbf{S} _i\cdot \mathbf{e}\mathbf )^2 \end{aligned}$$

where $J_{ij}$ is the exchange interaction between two spins $\mathbf{S} _i$ at the *i* site and $\mathbf{S} _j$ at the *j* site. The exchange interaction parameters, from the first to the third nearest neighbor atoms, were estimated by the constrained local moment approach in the VASP calculations. More detailed methodology is provided in Ref.^9^.

## Data Availability

The data that support the findings of this study are available from the corresponding author upon reasonable request.
